# Clinical significance of cyclin-dependent kinase inhibitor 2C expression in cancers: from small cell lung carcinoma to pan-cancers

**DOI:** 10.1186/s12890-022-02036-5

**Published:** 2022-06-24

**Authors:** Guo-Sheng Li, Gang Chen, Jun Liu, Deng Tang, Jin-Hua Zheng, Jing Luo, Mei-Hua Jin, Hua-Song Lu, Chong-Xi Bao, Jia Tian, Wu-Sheng Deng, Jing-Wei Fu, Yue Feng, Neng-Yong Zeng, Hua-Fu Zhou, Jin-Liang Kong

**Affiliations:** 1 Ward of Pulmonary and Critical Care Medicine, Department of Respiratory Medicine, The First Affiliated Hospital of Guangxi Medical University, No. 6 Shuangyong Road, Nanning, 530021 Guangxi Zhuang Autonomous Region People’s Republic of China; 2 Department of Cardiothoracic Surgery, The First Affiliated Hospital of Guangxi Medical University, Nanning, Guangxi Zhuang Autonomous Region People’s Republic of China; 3 Department of Pathology, The First Affiliated Hospital of Guangxi Medical University, Nanning, Guangxi Zhuang Autonomous Region People’s Republic of China; 4 Department of Pathology, The Affiliated Hospital of Guilin Medical University, Guilin, Guangxi Zhuang Autonomous Region People’s Republic of China; 5 Department of Respiratory and Critical Care Medicine, The Second People’s Hospital of Qinzhou, Qinzhou, Guangxi Zhuang Autonomous Region People’s Republic of China

**Keywords:** Cancer, Prognosis, Survival, Marker, Expression

## Abstract

**Background:**

Cyclin-dependent kinase inhibitor 2C (CDKN2C) was identified to participate in the occurrence and development of multiple cancers; however, its roles in small cell lung carcinoma (SCLC) remain unclear.

**Methods:**

Differential expression analysis of CDKN2C between SCLC and non-SCLC were performed based on 937 samples from multiple centers. The prognosis effects of *CDKN2C* in patients with SCLC were detected using both Kaplan–Meier curves and log-rank tests. Using receiver-operating characteristic curves, whether *CDKN2C* expression made it feasible to distinguish SCLC was determined. The potential mechanisms of *CDKN2C* in SCLC were investigated by gene ontology terms and signaling pathways (Kyoto Encyclopedia of Genes and Genomes). Based on 10,080 samples, a pan-cancer analysis was also performed to determine the roles of *CDKN2C* in multiple cancers.

**Results:**

For the first time, upregulated CDKN2C expression was detected in SCLC samples at both the mRNA and protein levels (*p* of Wilcoxon rank-sum test < 0.05; standardized mean difference = 2.86 [95% CI 2.20–3.52]). Transcription factor FOXA1 expression may positively regulate *CDKN2C* expression levels in SCLC. High *CDKN2C* expression levels were related to the poor prognosis of patients with SCLC (hazard ratio > 1, *p* < 0.05) and showed pronounced effects for distinguishing SCLC from non-SCLC (sensitivity, specificity, and area under the curve ≥ 0.95). *CDKN2C* expression may play a role in the development of SCLC by affecting the cell cycle. Furthermore, the first pan-cancer analysis revealed the differential expression of *CDKN2C* in 16 cancers (breast invasive carcinoma, etc*.*) and its independent prognostic significance in nine cancers (*e.g*., adrenocortical carcinoma). *CDKN2C* expression was related to the immune microenvironment, suggesting its potential usefulness as a prognostic marker in immunotherapy.

**Conclusions:**

This study identified upregulated CDKN2C expression and its clinical significance in SCLC and other multiple cancers, suggesting its potential usefulness as a biomarker in treating and differentiating cancers.

**Supplementary Information:**

The online version contains supplementary material available at 10.1186/s12890-022-02036-5.

## Introduction

Lung cancer is one of the most frequently detected cancers and the leading cause of cancer death worldwide [1, 2]. According to the estimated data for lung cancer, more than 2.20 million cancer cases were newly diagnosed, and 1.79 million cancer-related deaths occurred in 2020 globally [1]. Small cell lung cancer (SCLC) and non-small-cell lung cancer (NSCLC) are the main subtypes of lung cancer [3, 4]. Although the incidence of SCLC is lower than that of NSCLC, SCLC is characterized by rapid growth, easy metastasis, and unfavorable prognosis (e.g., the 2-year survival rate is just 15% in 2014 in America) [5]. Immune checkpoint inhibitor-related treatment was a promising direction for SCLC; however, its benefit was detected only in a few patients owing to a lack of biomarkers [6]. Therefore, discovering more new target genes associated with the development and progression of SCLC is crucial to provide potential avenues for early diagnosis and effective treatment regimens of SCLC.

The cyclin-dependent kinase inhibitor 2C (CDKN2C) protein, encoded by its homonymous gene (*CDKN2C*), is a member of the INK4 family. The CDKN2C protein can bind to CDK4 or CDK6 and reduces CDK kinase activation, contributing to cell cycle arrest in the G phase [7–9], where it was identified as a cell growth regulator. *CDKN2C* was identified to participate in the occurrence and/or development of and play essential roles in multiple cancers such as esophageal squamous cell carcinoma [10], liver hepatocellular carcinoma (LIHC) [8], and lung adenocarcinoma (LUAD) [11]. The gene was also reported to be related to the prognosis of several cancers, including sporadic medullary thyroid carcinoma (THCA) [12] and pancreatic neuroendocrine tumors [13]; thus, it was considered a potential biomarker of cancers. However, no reports about *CDKN2C* in SCLC have been previously published, resulting in a gap in the understanding of the expression and clinical significance of *CDKN2C* in SCLC, on which more research should focus.

In the present study, we assessed CDKN2C expression levels in SCLC and non-SCLC by using in-house data and data from Gene Expression Omnibus. The clinical significance of *CDKN2C* expression was elucidated, including its prognostic and differentiation effects. The underlying molecular mechanism of *CDKN2C* expression in SCLC was also discussed in the study. Furthermore, a pan-cancer analysis of *CDKN2C* expression and its clinical significance was performed to promote the understanding of the roles that *CDKN2C* plays in multiple cancers.

## Materials and methods

This study was approved by both the medical ethics review committee of the Affiliated Hospital of Guilin Medical University and the medical ethics review committee of the First Affiliated Hospital of Guangxi Medical University (2021[KY-E-246]). Informed consent was signed by all patients providing in-house samples.

### Collection of SCLC mRNA expression data and data normalization

SCLC-correlated datasets were obtained from multiple databases, namely ArrayExpress, Oncomine, Gene Expression Omnibus, and GDC Data Portal. The strategies used for screening datasets were “(mRNA or gene) AND (lung OR bronch*) AND (small cell).” The inclusion criteria were as follows: (1) samples from *Homo sapiens*; (2) lung/bronchus-related samples containing tissues/cells; and (3) data that include mRNA expression levels. The exclusion criteria were as follows: (1) datasets with duplicate and/or incomplete expression data and (2) samples from a merged dataset < 3.

Twenty-eight raw datasets (Additional file 1, 2) consisting of 379 SCLC samples and 533 non-cancer lung/bronchus samples were included in the study. After removing batch effects using the “sva” software package [14–17], datasets with the same platform were merged into one dataset (e.g., GPL570), and 13 merged datasets were generated (Additional file 1). Notably, among the 13 merged datasets, the dataset “GSE4824-GPL97” did not include the *CDKN2C* expression data and was only used for identifying the differential expression genes (DEGs). Moreover, 972 NSCLC samples of the 11 raw datasets were included (Additional file 2). *CDKN2C* mRNA expression levels were normalized with the “limma” package and log_2_ (*x* + 1) transformation.

### Collection of SCLC protein level data and immunohistochemistry experiment

Fifty-five in-house samples (26 SCLC samples and 29 non-cancer lung/bronchus samples) were obtained from the Affiliated Hospital of Guilin Medical University and the First Affiliated Hospital of Guangxi Medical University. An immunohistochemistry experiment was performed with these samples to detect differences in CDKN2C protein levels between the SCLC and control tissues. The anti-CDKN2C antibody (EPR15891, ab192239; Abcam Plc, Shanghai, China) was used in the experiment, which was conducted following the manufacturer’s instructions. Information on the experimental methods and protein staining scoring criteria were as described in our previous study [18]. Details of the CDKN2C protein level scoring criteria were as follows: for CDKN2C staining intensity scores: 0, 1, 2, and 3 represented no, light, moderate, and strong staining, respectively; for CDKN2C-positive staining cell number (percentage) score: 0, 1, 2, and 3 indicated ≤ 25%, 26–50%, 51%–75%, and > 75% positive staining cells. Ultimate CDKN2C protein levels were reflected by multiplying the staining intensity score with the percentage score. Clinical information of these samples is shown in Additional file 2.

### Clinical significance of *CDKN2C* expression in SCLC

The clinical significance of *CDKN2C* expression in SCLC was examined in the present study, including its effects on prognosis and cancer identification. A Kaplan–Meier curve was used to determine the overall survival (OS) difference between the high and low *CDKN2C* expression groups. The high and low *CDKN2C* expression groups were identified by the optimal threshold based on the maximally selected rank statistics of “survminer” package for R (v4.1.0). By using the “pROC” package [19–22], the area under the receiver-operating characteristic curve (AUC) was applied to evaluate both the usefulness of *CDKN2C* expression levels for distinguishing SCLC samples from non-SCLC samples and the accuracy of *CDKN2C* expression in differentiating SCLC samples from NSCLC specimens.

### Underlying mechanisms of *CDKN2C* expression in SCLC

Upregulated DEGs (Up-DEGs) were identified with the criteria – |log_2_ (fold change)|≥ 1 and standardized mean difference (SMD) > 1. *CDKN2C* positively related genes (CPRGs) were selected from genes with positive expression correlation with *CDKN2C* expression in > 35% (5/13) of the datasets. The predicted transcription factors (TFs) for *CDKN2C* expression were screened from the Cistrome Data Browser [23–27] with a cutoff score of > 0.9 (calculated using chromatin immunoprecipitation sequencing data). Matched sequences between predicted TFs and *CDKN2C* were identified by JASPAR [28] and Find Individual Motif Occurences [29]. The motif data of predicted TFs were obtained from JASPAR, and the seqlogo was drawn based on the “ggseqlogo” package. By using the “clusterProfiler” package [30–33], enrichment analyses were performed to explore the gene ontology terms and signaling pathways (Kyoto Encyclopedia of Genes and Genomes) of *CDKN2C* in SCLC. Gene ontology terms and signaling pathways with adjusted *p* values < 0.05 were selected in the study.

### Pan-cancer data collection

A pan-cancer dataset of The Cancer Genome Atlas and its clinical parameters were obtained from the Xena database (developed by the University of California, Santa Cruz). The sample inclusion criteria were as follows: samples from normal tissues, normal solid tissues, primary tumors, primary solid tumors, bone marrow, or primary blood-derived cancers. Samples of thirty-three cancers and twenty control tissues were selected for the pan-cancer analysis (Additional file 2). Four types of prognostic, namely OS, disease-specific survival (DSS), disease-free interval (DFI), and progression-free interval (PFI), were obtained from the Xena database. Clinical information of all samples included in the pan-cancer analysis is listed in Additional file 2.

### Collection of tumor mutation burden, microsatellite instability, and homologous recombination deficiency data

With the MuTect2 software [34–36] and “maftools” package, tumor mutation burden (TMB) was calculated on the basis of simple nucleotide variation data downloaded from the GDC portal. The process was completed using Sanger Box (v3.0). Microsatellite instability (MSI) and homologous recombination deficiency (HRD) data were from previous reports by Liu et al. [37] and Thorsson et al. [38] and downloaded from Sanger Box (v3.0).

### Immune microenvironment analyses

The relationship between *CDKN2C* expression and the immune microenvironment was examined using both the TIMER [39] and ESTIMATE [40] algorithms. The TIMER scores were calculated for evaluating the infiltration levels of six types of immune cells, namely B cell, CD4 T cell, CD8 T cell, neutrophil, macrophage, and dendritic cells. Three ESTIMATE scores (stromal, immune, and ESTIMATE scores) were also used to evaluate the correlation of *CDKN2C* with the immune microenvironment. TISIDB [41] was applied to investigate the association between *CDKN2C* expression and immune-related genes, including major histocompatibility complex molecules, immunoinhibitory genes, and immunostimulatory genes.

### Statistics analysis

Wilcoxon rank-sum tests and SMD were used to determine differential *CDKN2C* expression levels between SCLC and non-SCLC. A *p*-value > 0.1 in the *Begg*’s test indicated no significant publication bias in the SMD results. Kruskal–Wallis test was used for comparing *CDKN2C* expression levels between various cancer cell lines. The *Spearman* correlation coefficient was used to analyze the correlation of *CDKN2C* expression with TMB, MSI, HRD, TIMER scores, and ESTIMATE scores. Without specific identification, a *p*-value < 0.05 suggested statistical significance in all the statistical analyses.

**Fig. 1:**
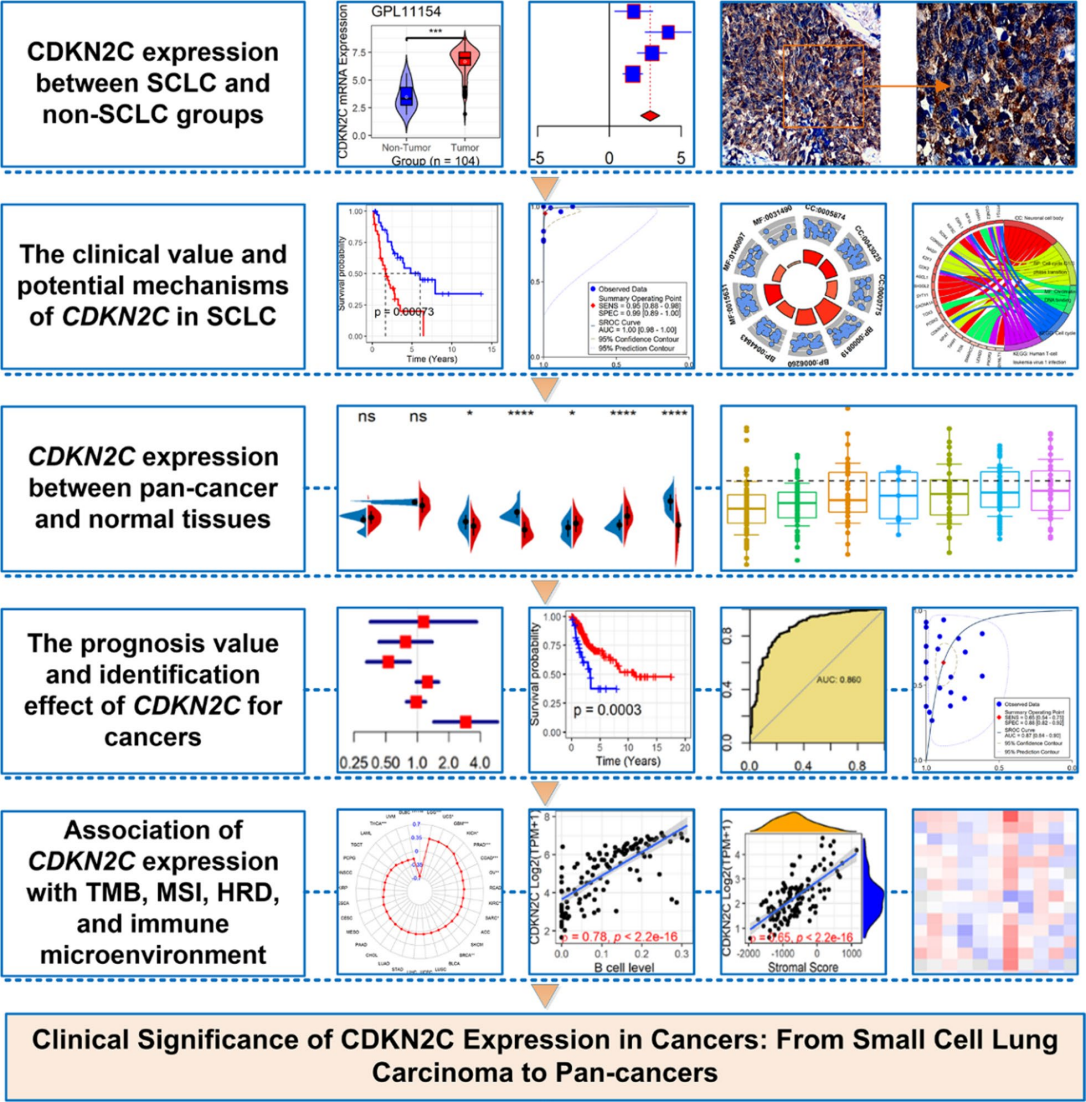
The design of this study. SCLC, small cell lung carcinoma; TMB, tumor mutation burden; MSI, microsatellite instability; HRD, homologous recombination deficiency

In this research, a series of packages [30, 42] of the R software (v4.1.0) were used to generate violin plots, forest plots, Kaplan–Meier curves, Cox regression forest plots, and receiver-operating characteristic curves. Figure 1 shows the design of this study.

## Results

### Upregulated *CDKN2C* expression in SCLC

**Fig. 2:**
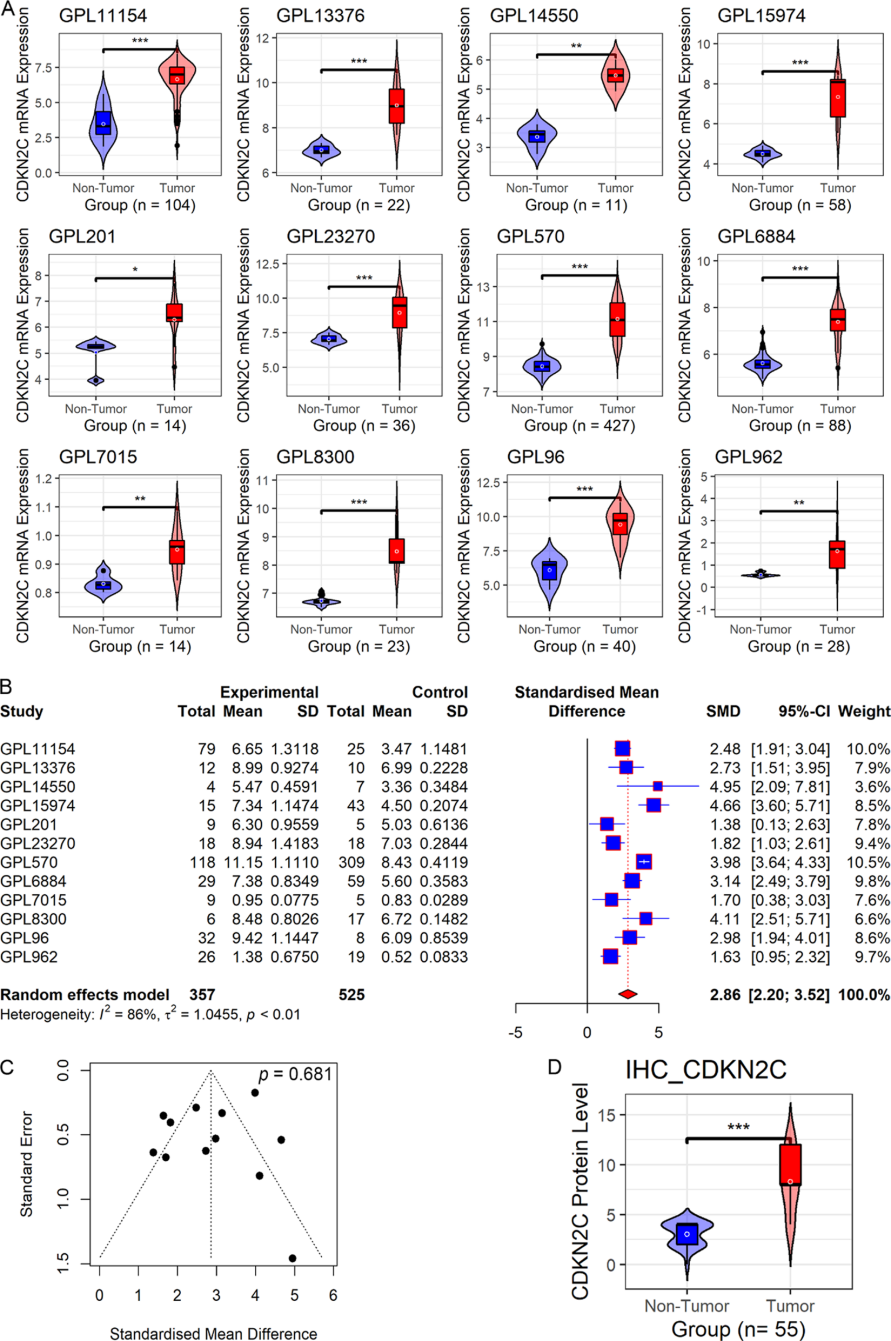
The expression of CDKN2C in small cell lung carcinoma (SCLC). Panel **A**: Violin plots of *CDKN2C* expression in SCLC. Panel **B**: A forest plot evaluating standard mean difference (SMD) of *CDKN2C* expression between SCLC and non-SCLC groups. Panel **C**: A funnel plot with *Begg*’s test for publication bias test. Panel **D**: A violin plot of CDKN2C protein levels between SCLC and non-SCLC groups

**Fig. 3:**
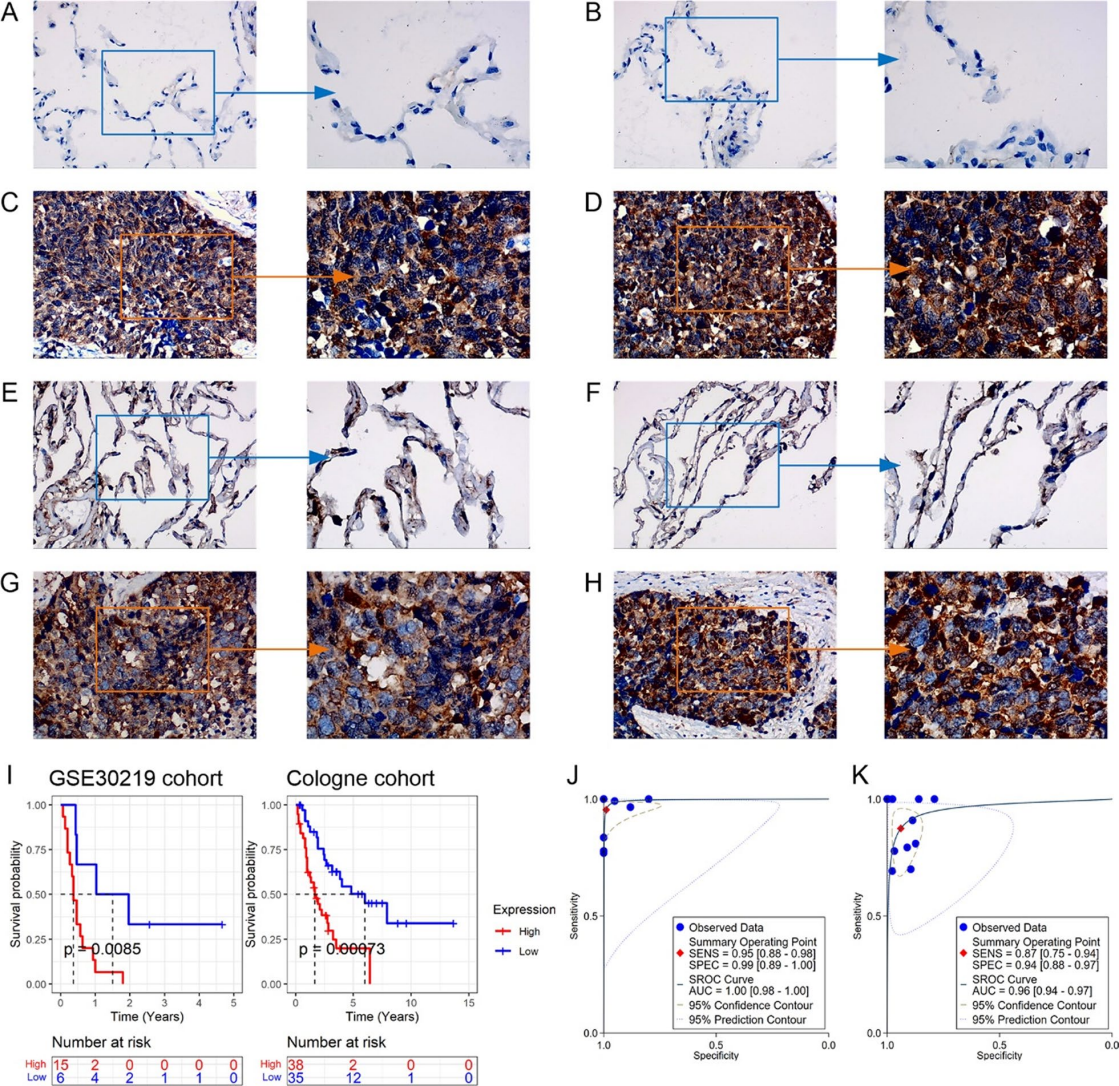
CDKN2C protein levels and the clinical significance of *CDKN2C* expression in small cell lung carcinoma (SCLC). Panels **A**–**H**: The protein levels of CDKN2C in non-SCLC (**A**, **B**, **E**, **F**) and SCLC (**C**, **D**, **G**, **H**) tissues under the microscope by in-house tissue microarrays. The left image of each two images is 200x, and the right image is 400x. Panel **I**: A Kaplan–Meier curve of overall survival between high- and low- *CDKN2C* expression groups. Panel **J**: Summary receiver operating characteristic curve for identifying SCLC from the healthy based on *CDKN2C* expression. Panel **K**: Summary receiver operating characteristic curve for identifying SCLC from the non-small-cell lung carcinoma based on *CDKN2C* expression. SENS, sensitivity; SPEC, specificity; AUC, area under the receiver-operating characteristic curve

Compared with the non-SCLC groups, the SCLC groups showed increased *CDKN2C* mRNA expression levels in 12 merged datasets (*p* values < 0.05; Fig. 2A). The finding was supported by the SMD results, as the SMD of each merged dataset and its 95% confidence interval were > 0 (Fig. 2B). Furthermore, high *CDKN2C* expression levels in the SCLC group were identified using the random-effects model (pooled SMD = 2.86; 95% confidence interval, 2.20–3.52; Fig. 2B). No significant publication bias was observed in the SMD results (*p* > 0.1; Fig. 2C). In the in-house samples, upregulated CDKN2C protein levels were detected in the SCLC tissues but not in the non-SCLC tissues (*p* < 0.05; Fig. 2D). On microscopic examination, positive CDKN2C staining was not conspicuous in the non-SCLC tissues (Fig. 3A, B, E, and F) unlike in the SCLC tissues (Fig. 3C, D, G, and H). Thus, upregulated CDKN2C expression at both the mRNA and protein levels was identified in SCLC.

### Significant clinical value of *CDKN2C* mRNA expression in SCLC

We attempted to exploit the clinical significance of *CDKN2C* mRNA expression in SCLC. As shown in Fig. 3I, based on the GSE30219 cohort, SCLC patients with higher *CDKN2C* mRNA expression levels had shorter overall survival times (*p* < 0.05). The result was supported by the other independent Cologne cohort [43] (Fig. 3I), suggesting the risk role of *CDKN2C* in the prognosis of patients with SCLC. Moreover, no difference in *CDKN2C* expression between various SCLC stages (*p* > 0.05; Additional file 3A), to some extent suggesting that the prognosis of *CDKN2C* mRNA expression in SCLC was not affected by SCLC stages.

*CDKN2C* mRNA expression levels well distinguished the SCLC samples from the non-SCLC samples, with a sensitivity, specificity, and AUC of ≥ 0.95 (Fig. 3J), demonstrating the conspicuous potential of *CDKN2C* expression levels to distinguish patients with SCLC from those without SCLC. Such a finding might provide clues for further research on the rapid screening of SCLC patients from the healthy based on detecting *CDKN2C* expression levels in human body fluids (e.g., blood). Moreover, based on the 11 raw datasets with both SCLC and NSCLC samples (*n* = 1135), *CDKN2C* expression made it feasible to differentiate SCLC from NSCLC (sensitivity = 0.87, specificity = 0.94, AUC = 0.96; Fig. 3K), implying *CDKN2C* was a potential marker identifying the two subtypes of lung cancer.

### Underlying mechanisms of *CDKN2C* expression in SCLC

**Fig. 4:**
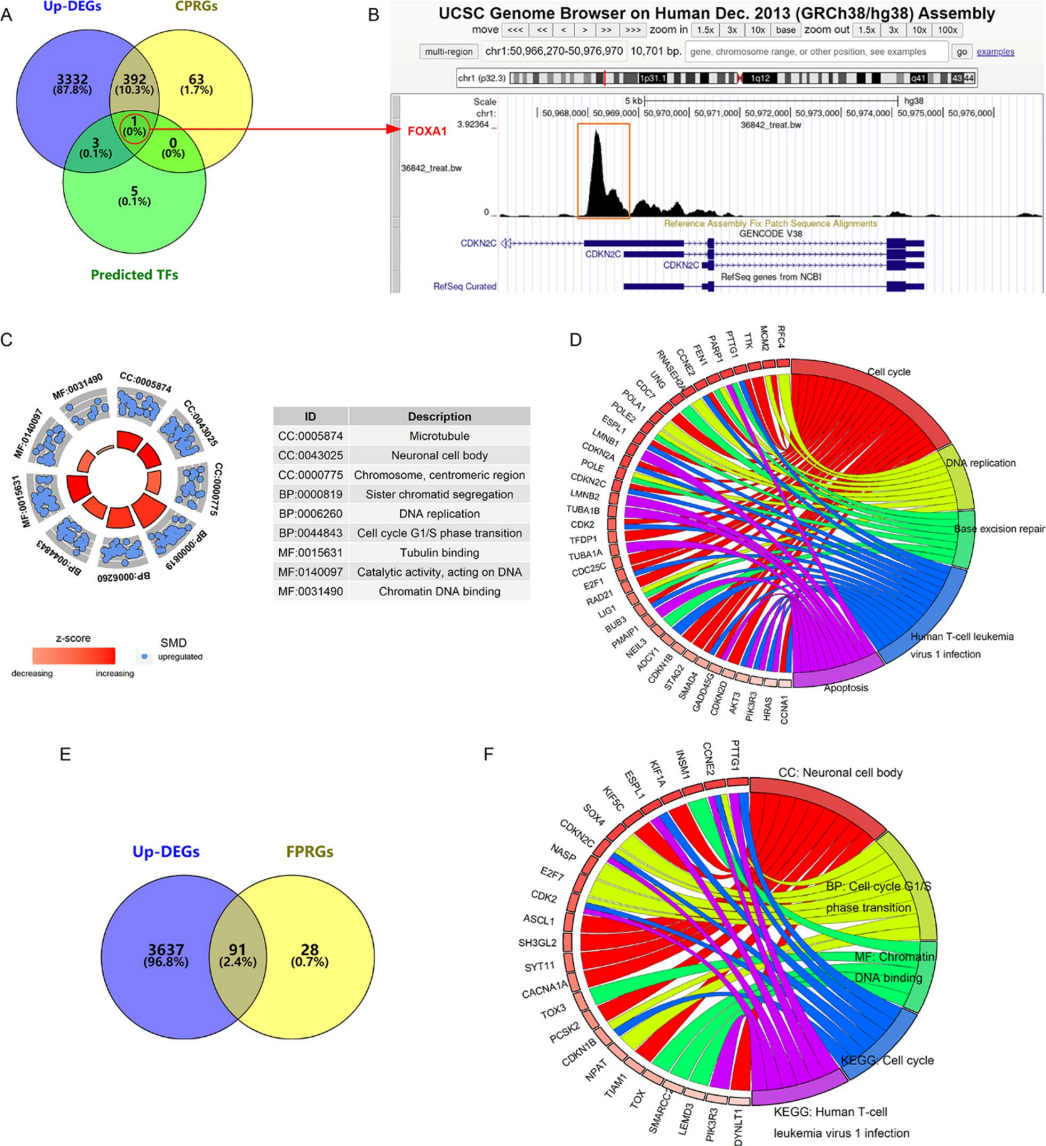
The potential mechanisms of *CDKN2C* and *FOXA1* in small cell lung carcinoma. Panel **A**: The Veen plot for screening predicted transcription factors for *CDKN2C*; Up-DEGs, upregulated differential expression genes; CPRGs, *CDKN2C* positively related genes. Panel **B**: For transcription factor FOXA1, binding sites exist with the potential promoter region of *CDKN2C*. Panel **C**: Gene ontology terms of *CDKN2C* positively related Up-DEGs. Panel **D**: Kyoto Encyclopedia of Genes and Genomes pathways of *CDKN2C* positively related Up-DEGs. Panel **E**: The Veen plot for screening *FOXA1* positively related Up-DEGs; FPRGs, *FOXA1* positively related genes. Panel **F**: Gene ontology terms and Kyoto Encyclopedia of Genes and Genomes pathways of *FOXA1* positively related Up-DEGs. CC, cellular component; BP, biological process; MF, molecular function

Concerning the conspicuous clinical value of *CDKN2C* in SCLC, we performed analyses to explore potential mechanisms of *CDKN2C* in the disease. In this research, 3,728 Up-DEGs and 456 CPRGs (including *CDKN2C*) were selected. Nine predicted TFs were screened using the Cistrome Data Browser. One potential TF (FOXA1) regulating *CDKN2C* expression was identified after the intersection of Up-DEGs, CPRGs, and predicted TFs (Fig. 4A). A chromatin-immunoprecipitation-sequencing peak (location: chr1:50967831–50968826; Additional file 3B) of FOXA1 (the motif shown in Additional file 3C) can be observed in the underlying promoter region, upstream of the transcription initiation site of *CDKN2C* (Fig. 4B), which supports the regulatory effects of FOXA1 on *CDKN2C*.

For the enrichment analyses, 392 *CDKN2C* positively related Up-DEGs were screened (Fig. 4A). These genes partly compose microtubules, neuronal cell bodies, and chromosome centromeric region (cell component) and are involved in sister chromatid segregation, DNA replication, and cell cycle G1/S phase transition (biological process) (Fig. 4C). They are associated with tubulin binding, catalytic activity (acting on DNA), and chromatin DNA binding (molecular function; Fig. 4C). *CDKN2C* positively related Up-DEGs cluster in multiple signaling pathways, including cell cycle, DNA replication, base excision repair, human T-cell leukemia virus 1 infection, and apoptosis (Fig. 4D). All results of enrichment analyses can be found in Additional file 4.

Given that FOXA1 may regulate *CDKN2C* in SCLC, we explored the underlying mechanisms of *FOXA1* in SCLC by *FOXA1* positively related Up-DEGs (Fig. 4E). As a result, *FOXA1* may affect the similar molecular mechanisms to *CDKN2C* in SCLC (Additional file 5), such as neuronal cell body (cell component), cell cycle G1/S phase transition (biological process), chromatin DNA binding (molecular function), cell cycle, and human T-cell leukemia virus 1 infection (signaling pathways) (Fig. 4F). Therefore, FOXA1 may regulate the expression of *CDKN2C* in SCLC and further influence the development of this disease by affecting the cell cycle (e.g., G1/S phase transition and DNA replication).

### Differentially expressed *CDKN2C* in pan-cancers

**Fig. 5:**
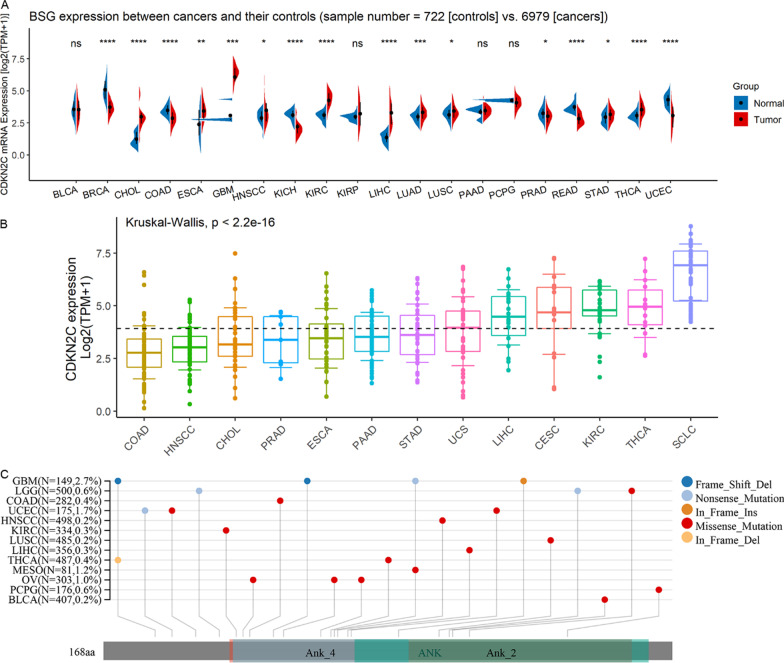
The expression of *CDKN2C* and its mutation landscape in pan-cancer. Panel **A**: The expression of *CDKN2C* mRNA expression in pan-cancer tissues. ^*^p < 0.05; ^ns^*p* ≥ 0.05. Panel **B**: The expression of *CDKN2C* mRNA expression in cancer cell lines. Panel **C**: The landscape of *CDKN2C*’s mutations in pan-cancer

Similar to SCLC, dysregulated *CDKN2C* expression was detected in 16 of the 20 cancers. Ten cancers had an upregulated *CDKN2C* expression, namely cholangiocarcinoma (CHOL), esophageal carcinoma (ESCA), glioblastoma multiforme (GBM), head and neck squamous cell carcinoma (HNSCC), kidney renal clear cell carcinoma (KIRC), LIHC, LUAD, lung squamous cell carcinoma (LUSC), stomach adenocarcinoma (STAD), and THCA (*p* < 0.05; Fig. 5A). Six cancers had downregulated *CDKN2C* expressions, namely breast invasive carcinoma (BRCA), colon adenocarcinoma (COAD), kidney chromophobe (KICH), prostate adenocarcinoma (PRAD), rectum adenocarcinoma (READ), and uterine corpus endometrial carcinoma (UCEC) (*p* < 0.05; Fig. 5A). Differentially expressed *CDKN2C* was also detected in 13 cancer cell lines, namely CESC (cervical squamous cell carcinoma and endocervical adenocarcinoma), CHOL, COAD, ESCA, HNSCC, KIRC, LIHC, pancreatic adenocarcinoma, PRAD, SCLC, STAD, THCA, and UCS (uterine carcinosarcoma) (Fig. 5B). The statistical *p*-values for *CDKN2C* expression differences between any two of the 13 cell lines can be consulted in Additional file 6.

### Mutation landscape of *CDKN2C* in pan-cancers

The mutations of *CDKN2C* can be observed in 13 cancers (GBM, etc.), and missense mutation was predominant (Fig. 5C). Among the 13 cancers, most mutations (2.7%) were found in GBM (Fig. 5C).

### Conspicuous clinical value of *CDKN2C* expression level in the prognosis and identification of pan-cancers

**Fig. 6:**
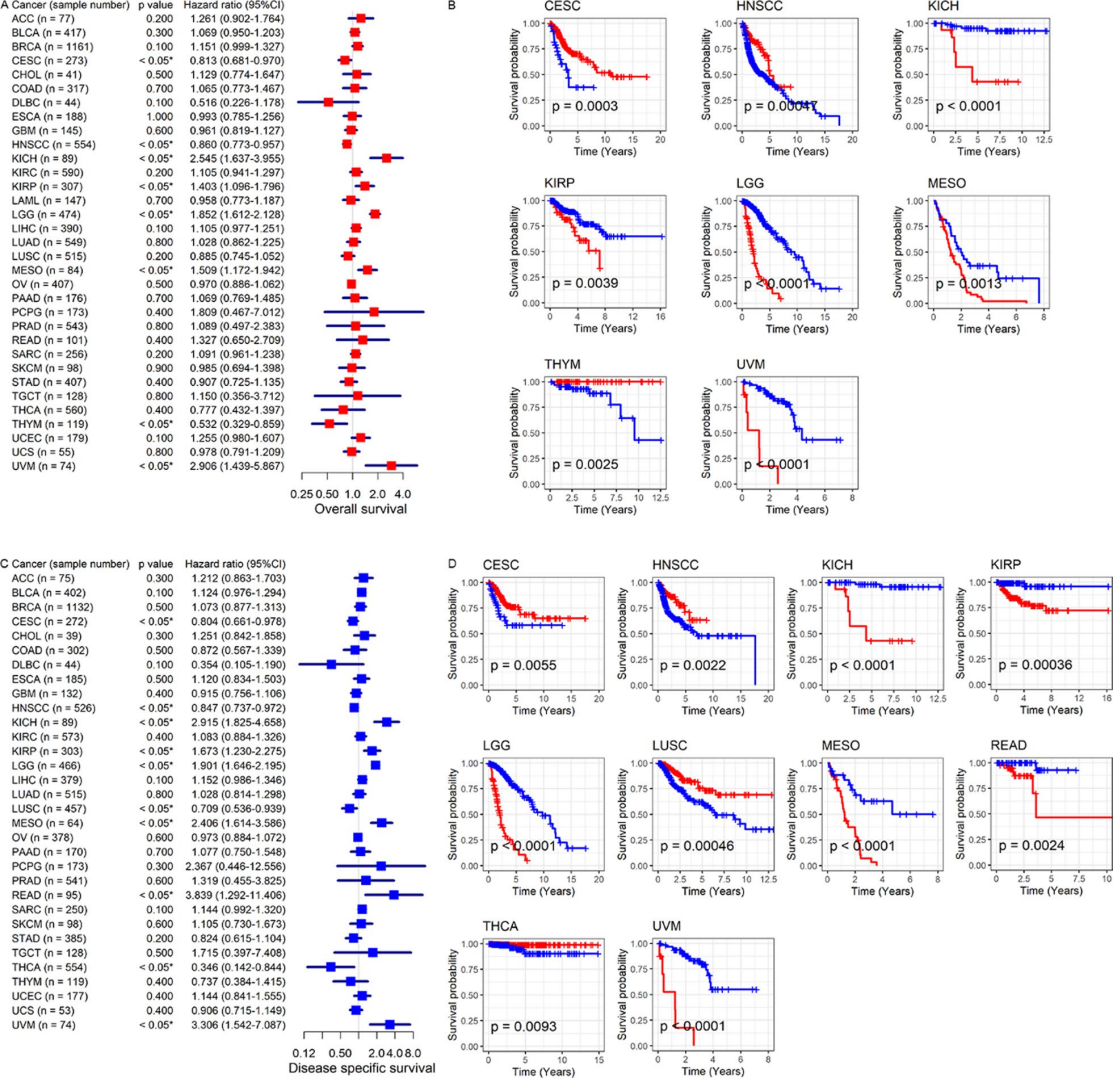
The correlation of *CDKN2C* expression with overall survival and disease-specific survival of cancer patients. Panels **A**, **B**: overall survival. Panels **C**, **D**: disease-specific survival. For panels **A** and **C**, *p*-values are based on univariate Cox gregression analysis; For panels **B** and **D**, *p*-values are based on log-rank tests

**Fig. 7:**
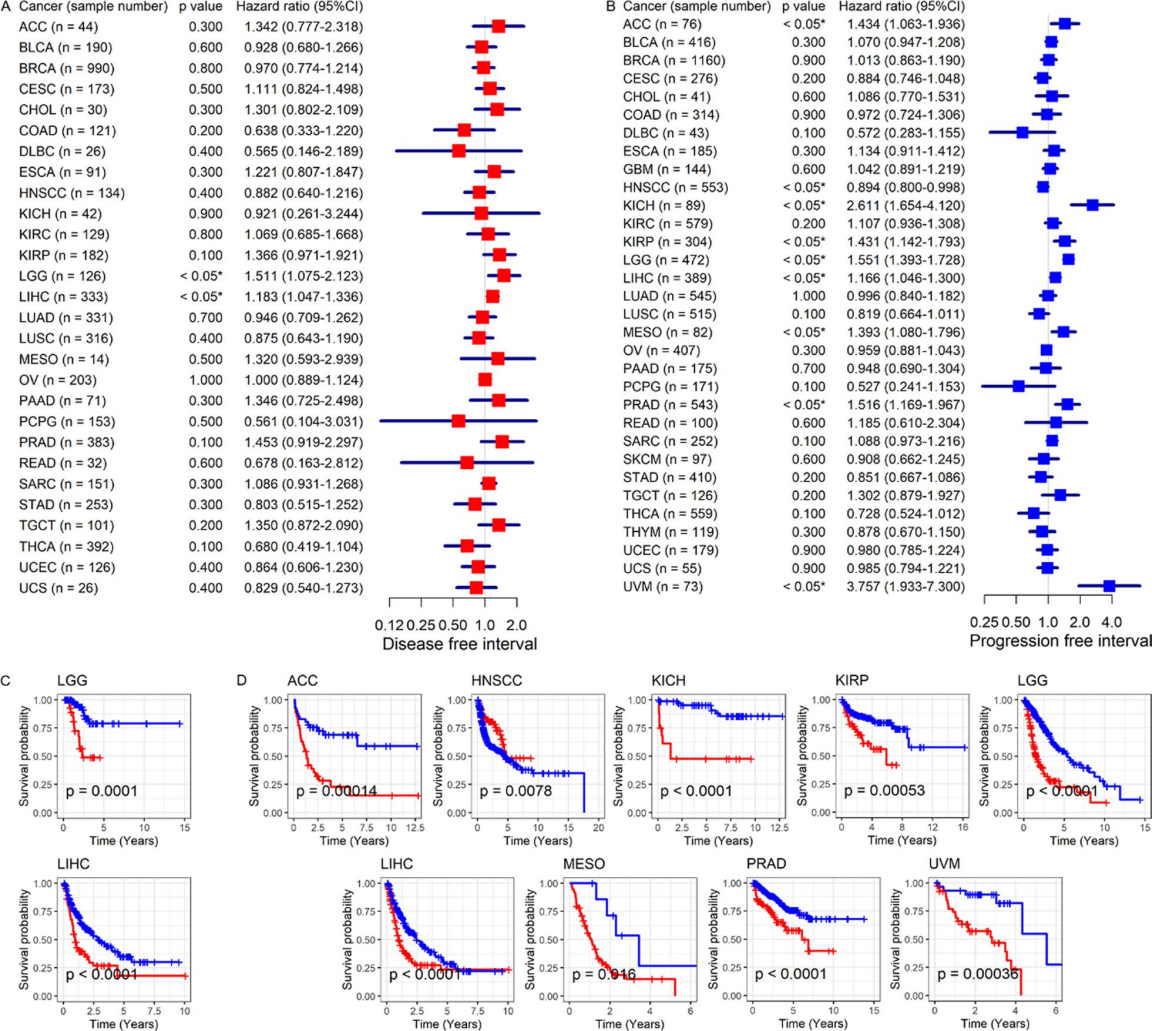
The correlation of *CDKN2C* expression with disease-free interval and progression-free interval of cancer patients. Panels **A**, **C**: disease-free interval. Panels **B**, **D**: progression-free interval. For panels **A** and **B**, *p*-values are based on univariate Cox gregression analysis; For panels **C** and **D**, *p*-values are based on log-rank tests

The independent prognosis value of *CDKN2C* in eight cancers was detected. Via univariate Cox regression, *CDKN2C* expression was related to poor OS in patients with KICH, KIRP (kidney renal papillary cell carcinoma), LGG (brain lower grade glioma), MESO (mesothelioma), and UVM (uveal melanoma) (hazard ratio > 1, *p* < 0.05) but was associated with favorable OS in patients with CESC, HNSCC, and THYM (Thymoma) (hazard ratio < 1, *p* < 0.05; Fig. 6A, B). Except for THYM, similar results can be observed for DSS (Fig. 6C, D). *CDKN2C* expression also represented a risk role for READ and a protective factor for LUSC and THCA (Fig. 6C, D). *CDKN2C* expression demonstrated an unfavorable DFI outcome in LGG and LIHC (Fig. 7A, C) and an unfavorable PFI outcome in ACC, KICH, KIRP, LGG, LIHC, MESO, PRAD, and UVM (Fig. 7B, D). In HNSCC, better PFI was detected in the patients with upregulated *CDKN2C* expression (Fig. 7B, D). Notably, based on the current clinical data of this study, multivariate Cox regression analysis indicated that *CDKN2C* expression was an independent prognosis (at least one of OS, DSS, DFI, and PFI) factor for eight of the 14 cancers listed above; and the eight cancers are CESC, KICH, LGG, LIHC, MESO, PRAD, THYM, and UVM (*p* < 0.05; Additional file 7). For the other six cancers, at least one of AJCC (American Joint Committee on Cancer) stage, age, and gender was an independent prognosis role for ACC, HNSCC, KIRP, and LUSC (*p* < 0.05); however, no independent prognostic factors were observed for READ and THCA (Additional file 7).

**Fig. 8:**
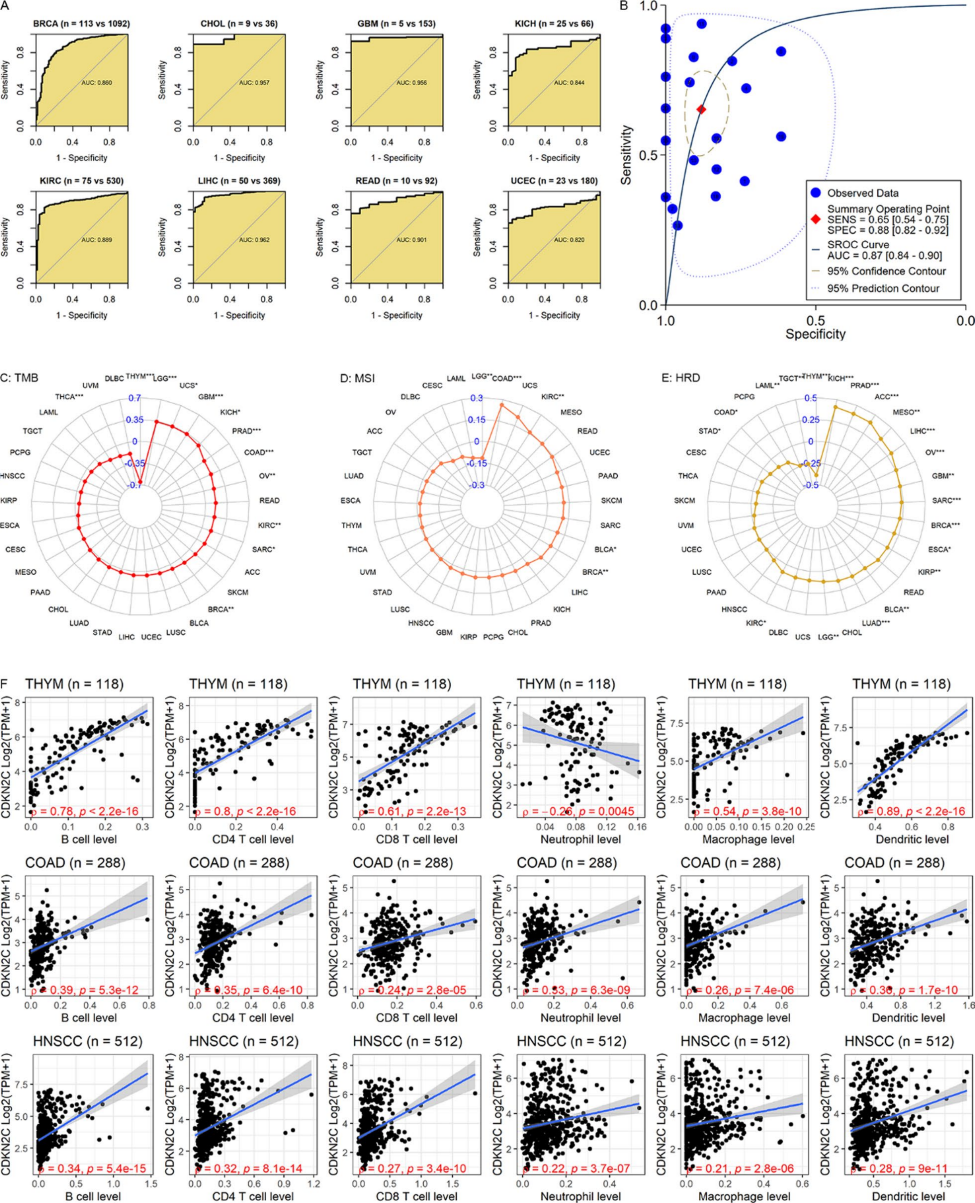
Effects of *CDKN2C* in identifying pan-cancers and the correlation between *CDKN2C* expression and immune microenvironment. Panels **A**, **B**: Receiver operating characteristic curves (panel **A**) and a summary receiver operating characteristic curve (panel **B**) for identifying cancers from non-cancers based on *CDKN2C* expression; AUC, area under the receiver-operating characteristic curve. Panels **C**–**F**: The associations of *CDKN2C* expression with tumor mutation burden (TMB), microsatellite instability (MSI), homologous recombination deficiency (HRD), and immune infiltration levels

AUC values (all > 0.8) of *CDKN2C* expression in eight of the 20 cancers indicated the conspicuous ability of *CDKN2C* in distinguishing the eight cancer tissues from the control tissues (Fig. 8A). The pooled AUC of 0.87 shown in Fig. 8B suggested that *CDKN2C* expression made it feasible to differentiate pan-cancer tissues from normal tissues.

### Association of *CDKN2C* expression with TMB, MSI, and HRD

TMB and MSI play important roles in the occurrence and/or progression of tumors and thus are considered cancer biomarkers [44]. In multiple tumors, HRD is one of the important indicators for treatment selection and prognostic evaluation [45]. As shown in Fig. 8C, *CDKN2C* expression was positively associated with TMB in LGG, UCS, GBM, and KICH (*Spearman ρ* > 0.3) and negatively associated with TMB in THYM (*Spearman ρ* = –0.66). *CDKN2C* expression was mildly related to MSI in COAD (*Spearman ρ* = 0.26; Fig. 8D). In five cancers (KICH, etc.), a positive correlation between *CDKN2C* expression and HRD was observed (*Spearman ρ* > 0.3; Fig. 8E).

### Correlation of *CDKN2C* expression with the immune microenvironment and immune-related genes

Among the 32 cancers with TIMER data (Fig. 8F and Additional file 8), *CDKN2C* expression represented weak to strong correlations with infiltration levels of all six types of immune cells in the top three cancers (i.e., all absolute values of *Spearman ρ* > 0.2, *p* < 0.05)—THYM, COAD, and HNSCC (Fig. 8F). In the three cancers, *CDKN2C* expression levels tended to be related to the increasing infiltration levels of six types of immune cells (except for neutrophils in THYM, Fig. 8F).

**Fig. 9:**
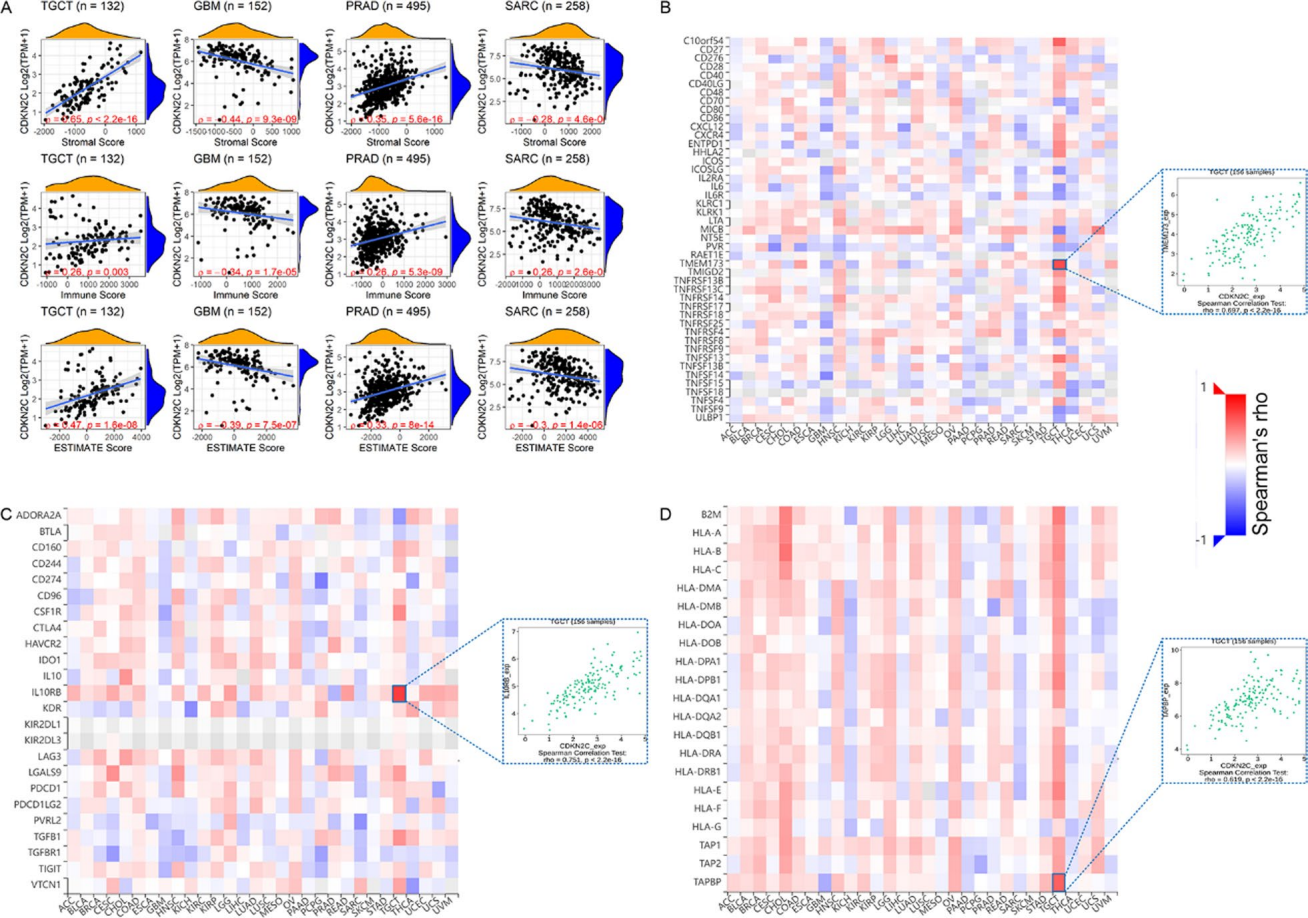
Correlation analyzes of *CDKN2C* expression with ESTIMATE scores (panel **A**) and immune-related genes (panels **B**–**D**)

According to the stromal, immune, and ESTIMATE scores (Fig. 9A and Additional file 9), *CDKN2C* expression was positively relevant to the immune microenvironment in TGCT (testicular germ cell tumor) and PRAD (all *Spearman ρ* > 0.25, *p* < 0.05) and negatively associated with the immune microenvironment in GBM and sarcoma (all *Spearman ρ* < –0.25, *p* < 0.05) (Fig. 9A).

In a few cancers (particularly TCGT), *CDKN2C* expression was related to multiple immune-related genes, including *TMEM173* (an immunostimulator) (Fig. 9B), *IL10RB* (an immunoinhibitory) (Fig. 9C), and *TAPBP* (a major histocompatibility complex molecule) (Fig. 9D) in TGCT. Such results indicated that *CDKN2C* might be involved in immune response, and its regulatory modes are different between cancer types and immune-related genes.

## Discussion

In this study, we identified upregulated *CDKN2C* expression and its clinical significance in the prognosis and identification of SCLC and other multiple cancers. By using 937 samples from multiple centers, upregulated CDKN2C expression was detected in SCLC samples at mRNA and protein levels. Transcription factor FOXA1 expression may contribute to increased *CDKN2C* expression levels in SCLC. High *CDKN2C* expression levels were related to the poor prognosis of patients with SCLC and showed conspicuous effects for distinguishing SCLC from non-SCLC, which has not been reported before. *CDKN2C* expression may play a role in the development of SCLC by affecting the cell cycle. Furthermore, on the basis of the first pan-cancer analysis of *CDKN2C*, the differential expression of *CDKN2C* and its prognostic significance were found in multiple cancers. Our research also demonstrates the correlation of *CDKN2C* expression with TMB, MSI, HRD, and immune microenvironment, suggesting its potential usefulness as a prognostic marker in immunotherapy.

*CDKN2C* has diverse expression patterns in different cancers. In our analyses of SCLC samples, high-*CDKN2C* mRNA expression levels were identified using 12 merged multicenter datasets, supported by the in-house dataset at protein levels. FOXA1 expression may contribute to the increased *CDKN2C* expression level in SCLC owing to the following: (1) similar to *CDKN2C*, it was an Up-DEG in SCLC; (2) it was positively associated with *CDKN2C* expression; and (3) the chromatin immunoprecipitation sequencing data support the finding. However, diverse *CDKN2C* expression patterns (compared with those in non-cancer tissues) were detected in various cancers as follows: upregulated *CDKN2C* mRNA expression in ten cancers (CHOL, ESCA, GBM, HNSCC, KIRC, LIHC, LUAD, LUSC, STAD, and THCA) and downregulated mRNA expression in six cancers (BRCA, COAD, KICH, PRAD, READ, and UCEC). There are some common features of *CDKN2C* expression in multiple cancers. That is, the high expression of *CDKN2C* was mainly observed in head and neck cancers (GBM and HNSCC), lung cancers (SCLC, LUAD, and LUSC), and digestive system cancers outside the colorectum (CHOL, ESCA, LIHC, and STAD). In contrast, the low expression of *CDKN2C* was found in the urinary system (KICH and PRAD) and colorectum (COAD and READ). However, the *CDKN2C* expression diversity in cancers is still apparent. For example, for renal cancers, upregulated *CDKN2C* expression was detected in KIRC, while the downregulated expression of *CDKN2C* was identified in the other subtype of renal cancer—KICH. The different *CDKN2C* expression patterns in diverse cancers may imply the inconsistent role of *CDKN2C* expression in different cancers.

*CDKN2C* plays dual roles in the prognosis of various cancers. Based on two independent cohorts in our study, the increased *CDKN2C* expression level is considered an indicator of poor prognosis in patients with SCLC; to our best knowledge, such a novel finding has not been reported before. For the pan-cancer analysis, *CDKN2C* expression represents a poor prognosis in seven cancers (LGG, etc.), while the gene is related to a favorable prognosis for CESC and THYM. Previously, in the gliomas-related research by Leone et al. [46], patients with p18 (encoded by *CDKN2C*)-positive oligodendrogliomas have reduced OS and PFI times, which is the same as our findings in LGG based on univariate Cox regression analysis and log-rank test. Moreover, we further identified the independent prognosis risk factor (not affected by AJCC stage, age, and gender) of *CDKN2C* expression in LGG in terms of OS, DSS, DFI, and PFI. For another cancer, Morishita et al. [47] demonstrated the association of p18 loss with poor prognosis in LIHC based on 51 samples; this may make sense, as p18 is known to arrest G0/G1 phase and thus *CDKN2C* presents a tumor suppressor gene for several cancers. However, with more samples (*n* = 341), we revealed that LIHC patients with upregulated *CDKN2C* expression had unfavorable prognoses (DFI and PFI), which is consistent with the results of Kong et al. [8]. Furthermore, as reported by Kong et al. [8], *CDKN2C* can promote the proliferative ability of LIHC cells and thus participates in the progression of LIHC. Therefore, whether the upregulated *CDKN2C* expression is a risk or protective factor for LIHC patients remains controversial and needs further investigation. Taking the current studies, we infer that: (1) the protective prognosis roles for some cancers (e.g., CESC and THYM) reflect the typical function of *CDKN2C* in suppressing tumors; (2) the mutational *CDKN2C* can promote cancer based on cell and mice experiments [8, 48, 49], which may be the reason for patients with elevated *CDKN2C* expression have poor prognosis in most cancers investigated in our study. Unfortunately, among the eight cancers where *CDKN2C* shows the independent prognostic effects, except for LGG and LIHC, no prognosis and corresponding mechanism research are available for reference. Collectively, the relationship between *CDKN2C* expression and patient prognosis may be complex, but an increased *CDKN2C* expression level was a prognosis risk signal for most cancers.

In addition to its prognostic effects, *CDKN2C* expression may be a marker for cancer identification and immunotherapy. *CDKN2C* expression showed its clear clinical value in distinguishing several cancers (particularly SCLC) from controls, implying its potential usefulness in screening cancers. To our best knowledge, this finding has not been obtained previously, indicating the novelty of our study. High TMB benefits patients with cancer during immunotherapy and thus was considered an immunotherapy biomarker [50]. MSI and HRD are also critical potential biomarkers for cancer treatment selection and prognostic evaluation [44, 45]. In our research, *CDKN2C* expression showed significant relevance with TMB, MSI, and HRD, suggesting the potential usefulness of *CDKN2C* expression level as a prognostic marker in immunotherapy, which is a worthwhile topic for further discussion.

The comprehensive molecular mechanisms of the effects of *CDKN2C* expression on cancers remain unknown. The suppressing effects of *CDKN2C* expression have been identified in numerous cancers due to its typical function. That is, through interaction with CDK4 or CDK6, CDKN2C protein participates in CDK kinase inactivation, subsequently blocking Rb phosphorylation and thus initiating cell cycle arrest from the G1 phase to the S phase [7, 10, 51]. *CDKN2C* expression may play a role in SCLC through its typical function, as *CDKN2C* positively related Up-DEGs cluster in cell components such as chromosome centromeric region, participate in the biological processes such as cell cycle G1/S phase transition, and are involved in molecular function catalytic activity (acting on DNA). This conclusion is also supported by the fact that these genes participate in signaling pathways such as the cell cycle and DNA replication. However, we believe that the mechanisms of *CDKN2C* expression in cancer (including SCLC) transcend its role in these signaling pathways because our pan-cancer analysis revealed that upregulated *CDKN2C* expression demonstrated poor prognosis in most cancers, including SCLC. Indeed, the result of our analysis confirmed that *CDKN2C* expression is a risk factor for multiple cancers for the following reasons: (1) decreasing *CDKN2C* expression (knockdown) levels did not consistently show a significant effect on cell cycle status [52]; (2) on the one hand, *CDKN2C* may stimulate the immune response and thus contribute to favorable prognosis for certain cancers (e.g., THYM); however, on the other hand, *CDKN2C* expression may block the cell cycle of not just cancer cells but also immune cells in some cancers based on our study (e.g., GBM and sarcoma) and previous research (e.g., acute promyelocytic leukemia) [53], whereas immune cells were an essential barrier against cancers; and (3) other functions of *CDKN2C*, such as that in cell differentiation, were identified in previous studies [54]. Taken together, the comprehensive molecular mechanisms of *CDKN2C* expression in cancers are complex and need further study.

The immune response may be one of the directions for studying *CDKN2C*. In our study, slight to moderate correlations of *CDKN2C* expression with immune cell infiltration levels, several immune scores, and immune-related gene expressions were observed in a few cancers. The findings initially suggested that *CDKN2C* may participate in the development and progression of cancer via the immune microenvironment, although this must be verified by experiments.

Our study has some limitations. First, the prognosis varies greatly depending on the clinical stage of SCLC patients; however, we did not analyze the difference in CDKN2C expression levels between the early stage and advanced stage resulting from sufficient stage data of SCLC patients. Second, we failed to collect body fluid samples to verify the potential of *CDKN2C* expression levels in differentiating cancer tissue from non-cancer tissue. Third, the pan-cancer analysis for exploring the roles of *CDKN2C* expression focused only on the mRNA levels of *CDKN2C*. Fourth, more efforts (e.g., experiments in vitro and in vivo) should be made to investigate the potential mechanisms of *CDKN2C* expression in cancers.

## Conclusion

Herein, upregulated *CDKN2C* expression and its clinical significance in SCLC were revealed using multicenter samples. The pan-cancer analysis of *CDKN2C* expression further demonstrated its prognostic and differentiation effects in multiple cancers. The findings and correlation of *CDKN2C* expression with TMB, MSI, HRD, and the immune microenvironment suggest its potential usefulness as a biomarker in treating and differentiating cancers.

## Supplementary Information

**Additional file 1:** The public datasets included for SCLC-related analyses.

**Additional file 2:** Clinical information of samples included in this study.

**Additional file 3:** CDKN2C expression difference between various SCLC stages (pane A), matched sequence of FOXA1 with CDKN2C (pane B), and FOXA1 motif (pane C).

**Additional file 4:** All results of enrichment analyses of CDKN2C positively related Up-DEGs.

**Additional file 5:** All results of enrichment analyses of FOXA1 positively related Up-DEGs.

**Additional file 6:** All p-values of Wilcoxon rank-sum tests of CDKN2C expression levels between various types of cell lines.

**Additional file 7:** Multivariate Cox regression analysis for detecting the independent prognosis significance of CDKN2C in pan-cancer.

**Additional file 8:** The associations of CDKN2C expression with immune infiltration levels.

**Additional file 9:** Correlation analyzes of CDKN2C expression with ESTIMATE scores.

## Data Availability

The datasets generated and/or analyzed during the current study are available in the Gene Expression Omnibus [https://www.ncbi.nlm.nih.gov/gds/], Depmap Portal [https://depmap.org/portal/download/], the Xena database [ http://xena.ucsc.edu/], and Sanger Box (v3.0) [http://vip.sangerbox.com/]. Direct persistent links for each public dataset and data on in-house tissue samples are available from Additional file 2.

## Abbreviations

SCLC: Small cell lung cancer
NSCLC: Non-small-cell lung cancer
CDKN2C: Cyclin-dependent kinase inhibitor 2C
LIHC: Liver hepatocellular carcinoma
LUAD: Lung adenocarcinoma
THCA: Thyroid carcinoma
DEGs: Differential expression genes
OS: Overall survival
AUC: Area under the receiver-operating characteristic curve
Up-DEGs: Upregulated differential expression genes
SMD: Standardized mean difference
CPRGs: CDKN2C positively related genes
TFs: Transcription factors
DSS: Disease-specific survival
DFI: Disease-free interval
PFI: Progression-free interval
TMB: Tumor mutation burden
MSI: Microsatellite instability
HRD: Homologous recombination deficiency
CHOL: Cholangiocarcinoma
ESCA: Esophageal carcinoma
HNSCC: Head and neck squamous cell carcinoma
KIRC: Kidney renal clear cell carcinoma
LUSC: Lung squamous cell carcinoma
STAD: Stomach adenocarcinoma
BRCA: Breast invasive carcinoma
COAD: Colon adenocarcinoma
KICH: Kidney chromophobe
PRAD: Prostate adenocarcinoma
READ: Rectum adenocarcinoma
UCEC: Uterine corpus endometrial carcinoma
CESC: Cervical squamous cell carcinoma and endocervical adenocarcinoma
UCS: Uterine carcinosarcoma
KIRP: Kidney renal papillary cell carcinoma
LGG: Brain lower grade glioma
MESO: Mesothelioma
UVM: Uveal melanoma
THYM: Thymoma
ACC: Adrenocortical carcinoma
AJCC: American Joint Committee on Cancer
TGCT: Testicular germ cell tumor
