# Supplementary figures and images for: Clinical significance of cyclin-dependent kinase inhibitor 2C expression in cancers: from small cell lung carcinoma to pan-cancers
Source: BMC Pulm Med. 2022 Jun 24;22:246. doi: 10.1186/s12890-022-02036-5 (PMC9233395; doi:10.1186/s12890-022-02036-5)

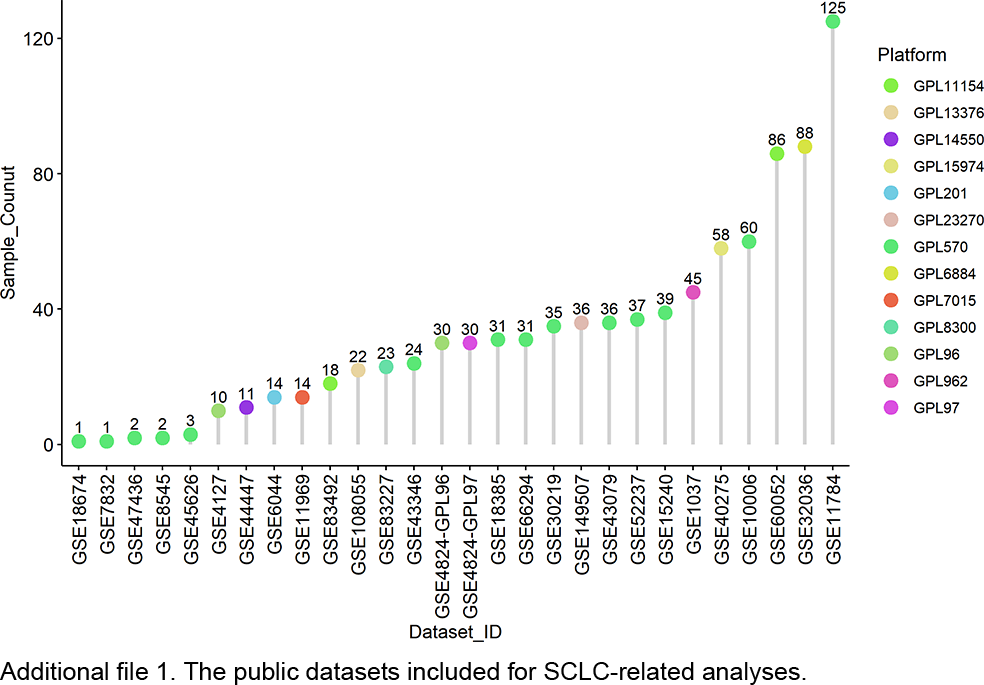

Supplement: Supplementary file 1 — Additional file 1: The public datasets included for SCLC-related analyses. [file 12890_2022_2036_MOESM1_ESM.tiff]

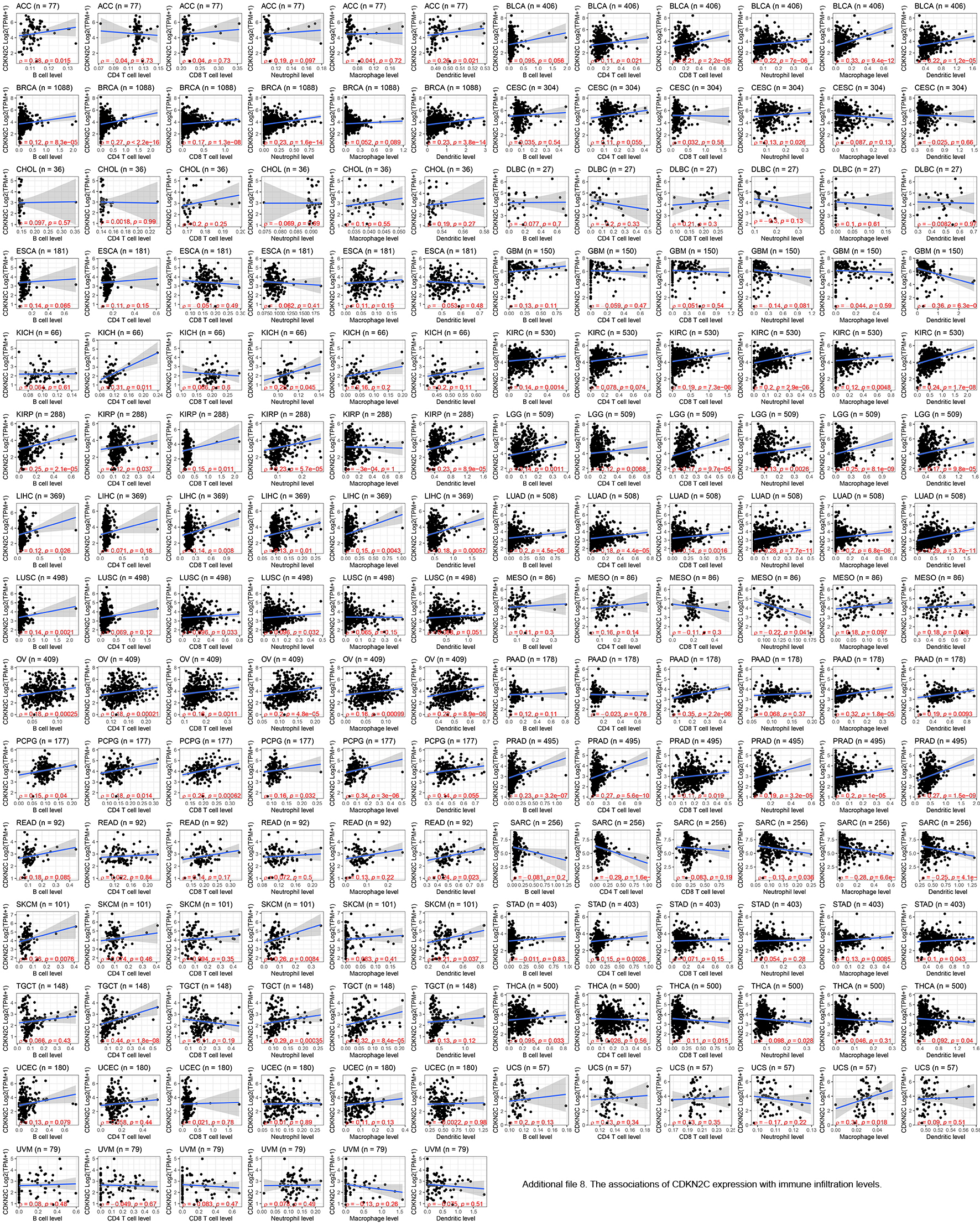

Supplement: Supplementary file 8 — Additional file 8: The associations of CDKN2C expression with immune infiltration levels. [file 12890_2022_2036_MOESM8_ESM.tif]

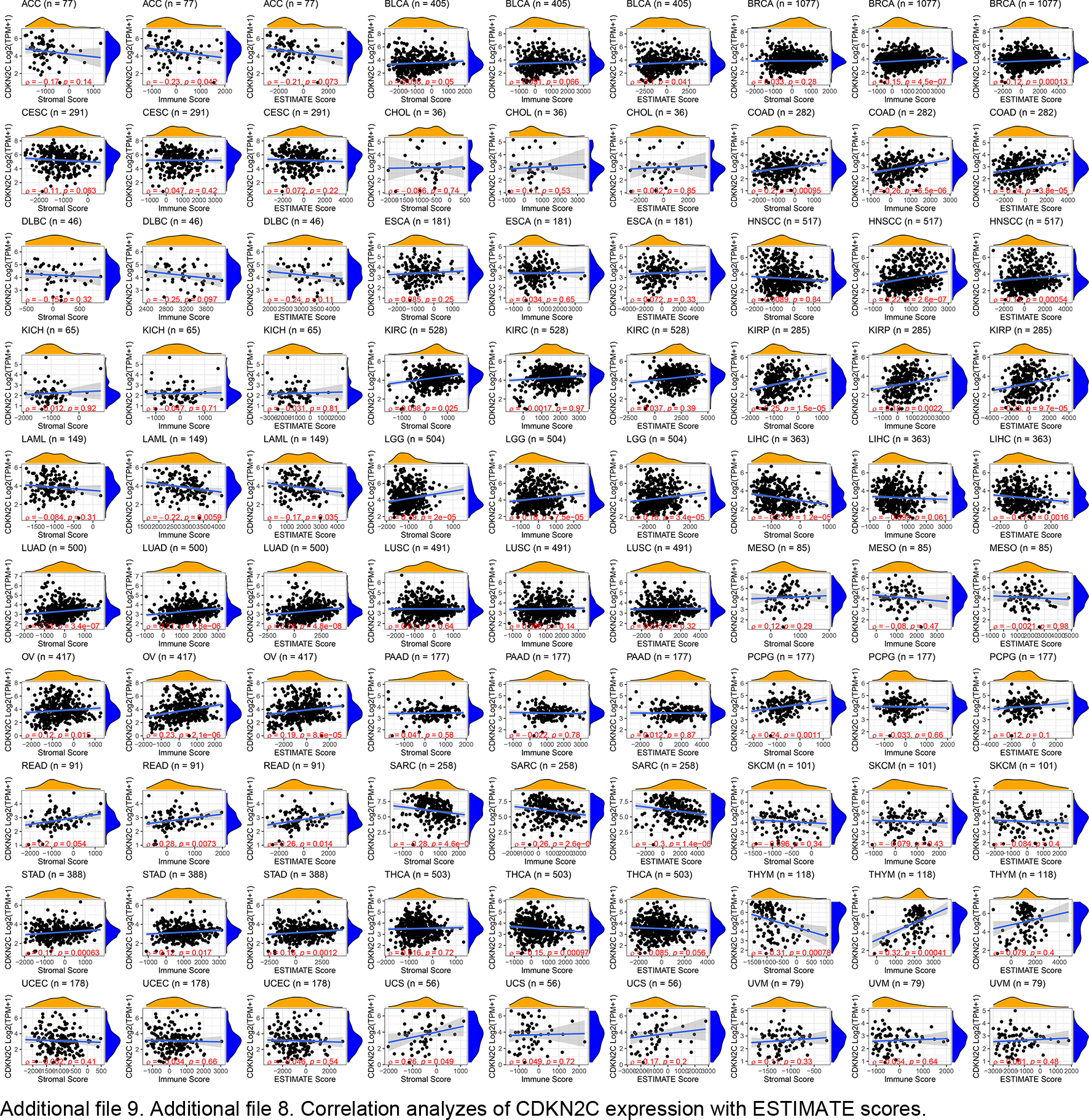

Supplement: Supplementary file 9 — Additional file 9: Correlation analyzes of CDKN2C expression with ESTIMATE scores. [file 12890_2022_2036_MOESM9_ESM.tif]
