# Supplementary material for: Clinical significance of cyclin-dependent kinase inhibitor 2C expression in cancers: from small cell lung carcinoma to pan-cancers
Source: BMC Pulm Med. 2022 Jun 24;22:246. doi: 10.1186/s12890-022-02036-5 (PMC9233395; doi:10.1186/s12890-022-02036-5)
Supplement: Supplementary file 3 — Additional file 3: CDKN2C expression difference between various SCLC stages (pane A), matched sequence of FOXA1 with CDKN2C (pane B), and FOXA1 motif (pane C). [file 12890_2022_2036_MOESM3_ESM.pdf]

A: No CDKN2C expression difference between various SCLC stages (III–IV vs. I–II).

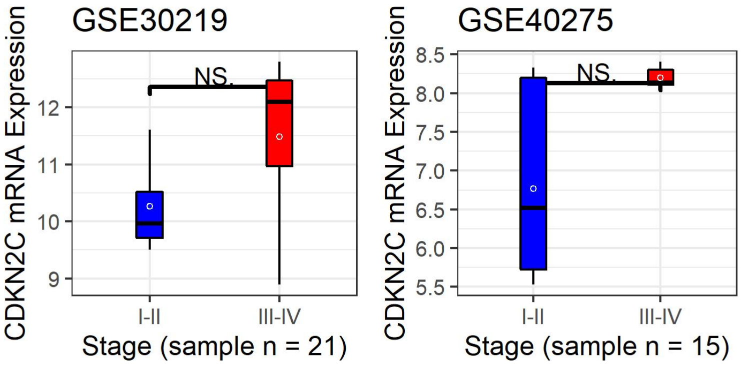

B: Details of the predicted matched sequence between FOXA1 and CDKN2C.

Tool	TF	Motif ID	Sequence	Start	Stop	Strand	Score[a]	*p*-value	Matched sequence
JASPAR[b]	FOXA1	MA0148.4	chr1:50967831-50968826	30	41	+	7.25	/	TAGAAAACACTT
FIMO[c]	FOXA1	MA0148.4	chr1:50967831-50968826	30	41	+	6.48	0.001	TAGAAAACACTT

Notes: [a]The score for the motif occurrence; [b]JASPAR (2022); [c]Find Individual Motif Occurences (Version 5.4.1).

C: The seqlogo of FOXA1 motif within FOXA1 ChIP-Seq peak.

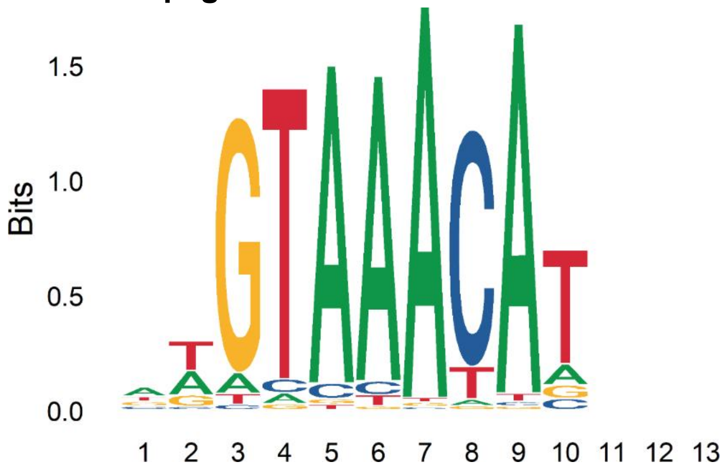

Additional file 3. CDKN2C expression difference between various SCLC stages (pane A), matched sequence of FOXA1 with CDKN2C (pane B), and FOXA1 motif (pane C).
